# The burden of mental disorder in Sierra Leone: a retrospective observational evaluation of programmatic data from the roll out of decentralised nurse-led mental health units

**DOI:** 10.1186/s13033-021-00455-1

**Published:** 2021-04-08

**Authors:** Helen Hopwood, Stephen Sevalie, Moshi Optat Herman, Dawn Harris, Katharine Collet, Abdulai Jawo Bah, Fenella Beynon

**Affiliations:** 1 King’s Sierra Leone Partnership, King’s Global Health Partnerships, King’s Centre for Global Health and Health Partnerships, School of Population and Environmental Sciences, King’s College London, Freetown, Sierra Leone; 2 34 Regimental Military Hospital, Freetown, Sierra Leone; 3 Kings College London Centre for Global Health and Health Partnerships, School of Population Health and Environmental Sciences, Faculty of Life Sciences and Medicine, King’s College London, London, UK; 4 School of Public Health, Imperial College London, London, UK; 5 Sustainable Health Systems, Freetown, Sierra Leone

## Abstract

**Background:**

In sub-Saharan Africa the treatment gap for mental disorders is high. In 2009, 98.0% of people with mental illness in Sierra Leone were not receiving treatment, partly due to the absence of public psychiatric facilities outside the capital. In response, the Ministry of Health and Sanitation rolled out nurse-led mental health units (MHU) to every district. This study aims to retrospectively evaluate the uptake of these services by examining the pathways to care, diagnosis, management, and treatment gap, to provide insight into the functioning of these units and the potential burden of mental health disorders in Sierra Leone.

**Methods:**

We evaluated the roll out of MHU using summary data from all units between 1 st January 2015 and 1 st January 2017, to establish the burden of diagnoses among service users, pathways to care, treatments provided, and treatment gaps. Negative binomial regressions examine bivariate relationships between diagnoses, treatments, and medication inaccessibility with demographics (age and sex), location (Freetown vs the rest and Ebola endemic regions vs the rest) and year.

**Results:**

We collected data from 15 MHU covering 13 districts in 24 months. There were 2401 referrals. The largest age category was 25–34 (23.4%). The prominent diagnoses were epilepsy (43.5%, associated with children) and psychosis (17.5%, associated with males). Reported depression (8.6%) and suicide attempts (33 patients) were low. Ebola endemic regions reported higher rates of grief, trauma, and medically unexplained symptoms. In 24.7% of cases where medication was required, it was not accessible.

**Conclusions:**

Nurse-led MHU can have a modest effect on the treatment gap in resource constrained environments such as Sierra Leone, particularly in epilepsy and psychosis.

## Background

Mental health and substance use disorders constitute a high burden of disease, accounting for 23% of disability-associated burden (years lived with a disability, YLD) globally and 19% in sub-Saharan Africa [1]. The treatment gap is highest in low- and middle-income countries (LMIC), ranging from 76.3 to 85.4%, and research on mental health services in these countries is limited [2, 3]. The gap is attributed to human resource constraints, stigma, weak technology and infrastructure, lack of political commitment, culture and traditional beliefs, and lack of research to guide policy formulation and implementation [4].

Task-sharing and health system restructuring have been shown to improve access to healthcare in non-communicable and infectious diseases [5, 6]. Evidence around task-sharing in mental health mostly focuses on primary care [7]. Although models of task-sharing with trained nurses have been implemented, there is little evidence about the effectiveness of these programs outside of primary care in Africa [8–10].

In Sierra Leone, there are no recent mental health prevalence studies; however, a 2002 Ministry of Health and Sanitation (MOHS) survey at the end of the civil war, indicated prevalence rates of 2% psychosis, 4% depression, 4% severe substance abuse, 1% mental retardation, and 1% epilepsy [11]. The prevalence of mental health problems is likely to have been adversely affected by the 1991–2002 civil war, economic problems, gender based violence, poor health outcomes including high rates of maternal and child mortality, and the recent Ebola epidemic [12–16]. In 2009, only 2058 of an estimated 102 000 people with severe mental illness received treatment, resulting in a treatment gap of 98.0% [11]. Until recently, the only MOHS treatment facility was the Sierra Leone Psychiatric Hospital (SLPH) in Freetown.

In response, the MOHS collaborated with partner organisations to train 21 nurses in a 12–18 month mental health program in 2012 [17, 18]. Of those trained, 19 remain in active service in addition to three nurses who completed training abroad. This created a mental health nurse workforce on par with the median for low-income countries (0.26 per 100,000 population) [19].

In 2015 these staff established Mental Health Units (MHU) in all 14 districts. A centralised Child and Adolescent (CAMH) Unit was established in 2016. Each MHU is positioned within the general hospital. They take referrals from any source and operate without strict eligibility criteria. The nurses assess and treat patients in accordance with the mental health Gap Action Plan (mhGAP) [20].

The availability of monitoring and evaluation (M&E) summary reports over 2 years provides an opportunity to establish the burden of disease among patients presenting to district nurse-led mental health services and range of treatments provided in decentralised MHU. The aim of the study was to use these reports to examine diagnoses, pathways to care, management and treatment gaps in the MHU. This analysis will give insight into the burden of mental illness in Sierra Leone and contribute towards understanding the mechanisms for addressing the mental health treatment gap in resource constrained environments.

## Methods

### Setting and participants

The MHU are clinics embedded within general government hospitals, including one general referral hospital, one children’s hospital, one military hospital, and 12 district hospitals. Medication supply is sporadic and largely relies on donations from non-governmental organisations (NGOs) [21]. Most MHU (13 of 15) are staffed by one nurse working alone. The clinics take referrals from the general hospital (both inpatient and outpatient), the community (including Primary Healthcare Units, NGOs, the Ministry of Social Welfare, Gender and Children’s Affairs, Ebola Survivor Units and the police), the Psychiatric Hospital and self-referrals by the patient or their families. The staff conduct outreach work to raise awareness about mental health disorders and create referral networks, for example by carrying out formal training sessions and radio campaigns. The referral criteria include signs of any mental illness, suicidality, intellectual disability or epilepsy. There are no criteria which might exclude a person from accessing the MHU.

### Data

Data were extracted from monthly M&E reports which were submitted by each of the 15 MHU to an NGO providing support to the MHU between 1st January 2015 and 1st January 2017. Each report included summary data for the month on new and follow-up patients (patients that were already known to the clinic prior to that month), including basic sociodemographic details, diagnoses, referral sources, and interventions (psychological, social, or psychotropic medication) (Appendix 1). The diagnostic categories of the mhGAP including mental, neurological and substance misuse disorders were used as opposed to ICD or DSM criteria because this aligned with the MHU staff’s training; furthermore, the mhGAP has been designed to simplify clinical management in resource-constrained environments [20]. Where reports were missing for a given month, these were retrospectively filled from clinic registries.

### Analysis

The analysis begins with a descriptive summary of total monthly records including patients’ demographics, diagnoses, pathways to care, treatments, treatment gaps, differences across facilities and missing data. Additional analysis identified statistically significant differences using bivariate regressions in total monthly counts of diagnoses and treatments by patients’ demographic characteristics (age groups and total number by sex), location in Freetown vs other districts, location in areas with high Ebola endemicity (Freetown, Kenema, Bombali, and Kailahun) vs low Ebola endemicity, and time (2015 vs 2016). The aggregate monthly counts and subsequent proportions were summed by facility. There was one clinic per district other than Freetown which had three facilities, whose counts were combined to examine variations across districts. We used negative binomial regressions because the outcome variables (total monthly counts per clinic of diagnoses and treatments) took non-negative integer values. Series of bivariate negative binomial regressions estimated counts of diagnoses (epilepsy/seizures, substance abuse, intellectual disability, psychosis, depression, other, and medically unexplained somatic complaints) and interventions (psychological, social, medication, medication not available, and medication unaffordable). The total monthly caseload was used as the exposure variable. Analysed data encompass new patients—to describe the characteristics of people accessing the service—except in the case of interventions, which includes new and follow-up patients to provide insight into the range of treatments that can be provided on an on-going basis.

## Results

Over the 2-year period, 285 data sheets were generated. The total number of new patients reported was 2401. Some reports were found to have missing data which could not be retrospectively filled from clinic records. The median proportion of available data per category of the data sheet was 70.9% (IQR 64.4–86.3%), with more data missing from certain categories (sex, age, diagnosis, social intervention and medication not affordable) and certain MHUs (Kenema, Koidu, Magburaka and Moyamba), as shown in Appendix 2.

### Patient demographics and diagnoses

For the available data, sex was recorded for 1045 patients, of whom 51.5% (538) were female.

**Fig. 1:**
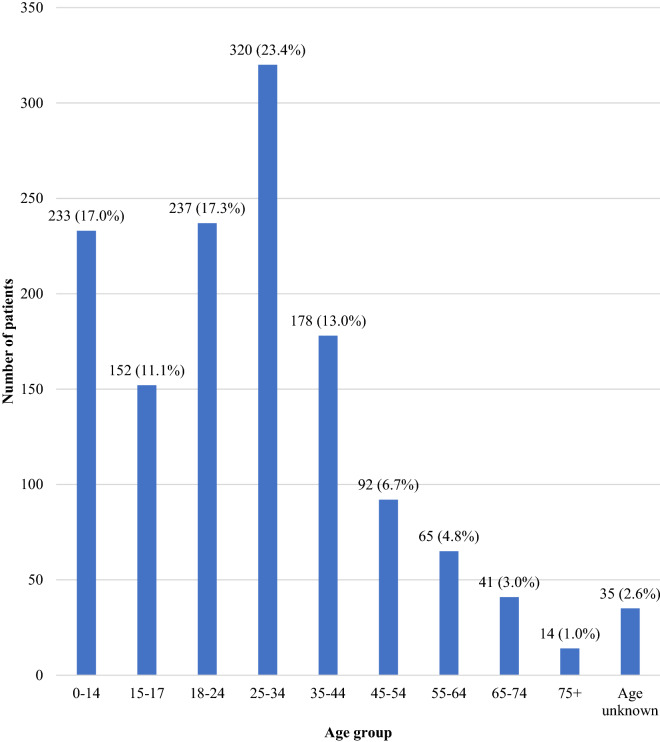
Age groups—number of patients referred to all mental health units in 2015 and 2016; by age group

68.9% (942 of 1367) of patients were aged between 0 and 34 and 17.0% (233) were under 15 (Fig. 1).

**Fig. 2:**
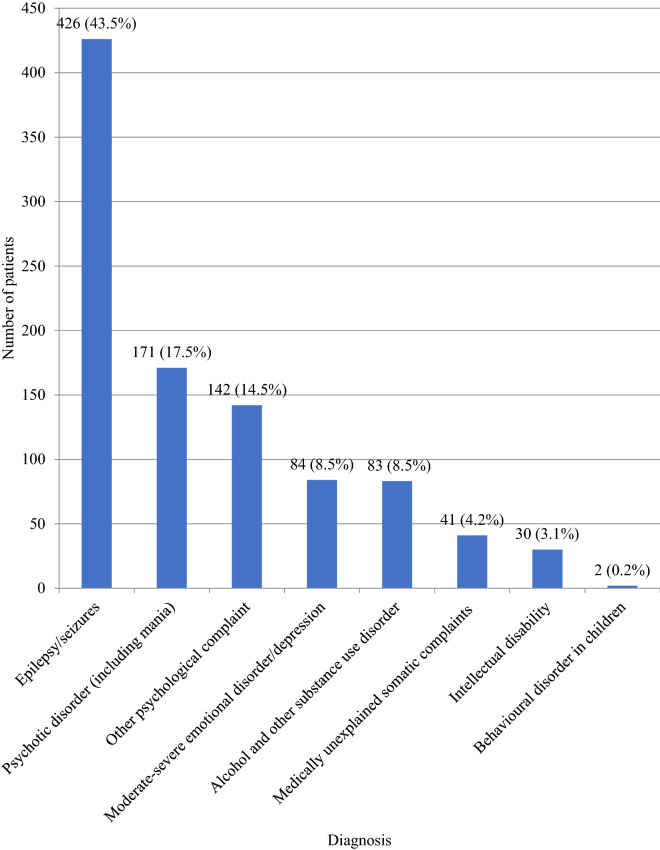
Diagnosis groups—number of patients referred to all mental health units in 2015 and 2016; by diagnosis group according to the mhGAP

Epilepsy/seizures were most frequently diagnosed (43.5%, 426 of 979), followed by psychosis (17.5%, 171) and ‘other psychological complaints’ (14.5%, 142), which is a diagnosis of exclusion if no other mental health disorder is present, encompassing stress, grief and trauma. Moderate-severe depression (8.6%, 84) was seen less commonly (Fig. 2).

The number of patients in age group 0–14 was positively associated with the number of epilepsy diagnoses (p < 0.0001, Table 1). Intellectual disability diagnoses were positively associated with age group 0–14 (p = 0.008) and negatively associated with groups 25–34 (p < 0.0001) and 35–44 (p = 0.004). Psychotic disorders showed a positive relationship with age groups 25–34 (p = 0.05) and age 75 and above (p = 0.04). Depression was positively associated with age group 55–64 (p = 0.02).

**Table 1:** Comparison of the number in each diagnosis category with different demographic characteristics and year—relationships between the number with each diagnosis and selected independent variables (number in each group: age, sex, residence within Freetown vs the rest, residence within districts with high Ebola endemicity vs the rest, and year of first presentation 2015 vs 2016) using bivariate negative binomial regressions *p < 0.05

	Epilepsy/seizures		Alcohol and other substance use disorder		Intellectual disability		Psychotic disorder (including mania)		Moderate-severe emotional disorder/depression		Other psychological complaint		Medically unexplained somatic complaints	
	Incidence rate ratio	p-value	Incidence rate ratio	p-value	Incidence rate ratio	p-value	Incidence rate ratio	p-value	Incidence rate ratio	p-value	Incidence rate ratio	p-value	Incidence rate ratio	p-value
Age groups														
0–14	1.17*	0.00016	0.88*	0.0091	1.13*	0.0081	0.94	0.066	0.91*	0.014	0.91*	0.042	0.85*	0.039
15–17	0.93	0.14	1.11	0.066	1.08	0.16	1.02	0.60	1.03	0.54	1.06	0.38	1.05	0.60
18–24	1.04	0.21	1.00	0.98	1.14*	0.0038	0.98	0.38	1.05	0.097	1.00	0.93	1.08	0.17
25–34	0.95*	0.042	1.03	0.28	0.88*	< 0·0001	1.04*	0.049	1.00	0.86	1.01	0.72	1.02	0.68
35–44	1.02	0.57	0.98	0.54	0.89*	0.0044	1.02	0.36	1.03	0.37	1.10	0.088	0.98	0.78
45–54	1.00	0.94	0.98	0.81	0.93	0.27	1.04	0.37	1.04	0.44	1.05	0.56	1.05	0.61
55–64	0.87	0.099	0.92	0.27	0.87	0.11	0.96	0.46	1.17*	0.020	1.01	0.95	1.36*	0.0059
65–74	1.10	0.36	0.91	0.32	1.09	0.39	0.88	0.067	1.11	0.20	0.95	0.73	0.93	0.59
75+	0.93	0.65	1.09	0.67	0.91	0.69	1.39*	0.037	0.92	0.62	1.05	0.84	0.79	0.36
Unknown	1.25	0.10	0.72	0.069	0.92	0.62	0.80	0.099	0.75*	0.046	1.00	0.99	1.41	0.20
Constant	0.16*	0	0.12*	0	0.03*	0	0.20*	0	0.03*	0	0.07*	0	0.02*	0
N	238		239		237		239		233		239		239	
Sex														
Number of males	1.04	0.11	1.05*	0.022	0.99	0.67	1.06*	0.0026	1.03*	0.011	0.99	0.29	0.98	0.31
Number of females	1.00	0.84	0.93*	< 0·0001	1.02	0.64	0.95*	0.00017	1.01	0.54	1.03	0.070	1.03	0.13
Constant	0.11*	0	0.12*	0	0.06*	0	0.21*	0	0.03*	0	0.08*	0	0.03*	0
N	215		216		214		216		212		216		216	
Freetown vs the rest														
Freetown (ref: the rest)	0.03*	0	0.23*	< 0.0001	0.31*	0.0085	0.29*	< 0.0001	0.72	0.33	0.85	0.71	0.26*	0.013
Constant	0.31*	0	0.11*	0	0.05*	0	0.22*	0	0.06*	0	0.09*	0	0.04*	0
N	245		246		244		246		240		246		246	
Ebola—districts with high endemicity vs the rest														
High Ebola endemicity districts (ref: the rest)	0.68	0.071	0.91	0.72	0.33*	0.00019	0.67*	0.031	1.01	0.95	2.42*	0.00045	2.65*	0.0047
Constant	0.32*	0	0.10*	0	0.07*	0	0.24*	0	0.05*	0	0.05*	0	0.02*	0
N	245		246		244		246		240		246		246	
2015 vs 2016														
2015 (ref: 2016)	0.72	0.23	1.45	0.23	1.03	0.93	0.96	0.87	2.74*	< 0.0001	1.78	0.069	3.77*	0.00018
Constant	0.35*	< 0.0001	0.06*	0	0.06*	0	0.19*	0	0.02*	0	0.05*	0	0.01*	0
N	175		175		173		175		175		175		175	

The incidences of psychosis (p = 0.003), depression (p = 0.01) and substance abuse (p = 0.02) were positively associated with the number of males (Table 1). All other diagnoses were not associated with sex.

A total of 33 patients presented to services having attempted suicide or self-harm (1.4% of 2401).

### Patient numbers and pathways to care

The median number of new patients per open clinic per month was 4.0 (IQR 1.0–10.0), and the total number per clinic per month ranged from 0 to 93.

91.7% (1398 of 1524) were outpatients and the remainder were inpatients in the general hospital. The most common referral sources were self (39.2%, 661 of 1686) and another department within the hospital (33.0%, 556). 17.6% (297) patients were from ‘other’ sources including NGOs, Primary Healthcare Units, and the police. 8.4% (141) were from Ebola Survivor Clinics, 1.8% (30) were referred by the Ministry of Social Welfare, Gender and Children's Affairs, and one patient was referred by the Psychiatric Hospital.

### Mental health treatments

The median number of contacts (new and follow-up) per clinic per month was 15.0 (6.0–38.0). Across the study period there were 4160 psychological interventions, 2800 psychotropic prescriptions, and 1118 social interventions. The median number of psychological interventions per clinic per month was 6.0 (IQR 1.0–18.0). Comparative figures for psychotropic prescriptions were 3.0 (0.0–13.5) and for social interventions 0.0 (0.0–4.0).

Social interventions were positively associated with age group 0–14 (p = 0.001) and negatively associated with age groups 35–44 (p = 0.006) and 45–54 (p = 0.002, Table 2). Medication and psychological interventions were less strongly associated with age.

**Table 2:** Comparison of the number of each category of intervention given with different demographic characteristics and year—relationships between the number provided with each treatment (psychological, social, medication, and those for whom medication with unavailable or unaffordable) and selected independent variables (number in each group: age, sex, residence within Freetown vs the rest, residence within districts with high Ebola endemicity vs the rest, and year 2015 vs 2016) using bivariate negative binomial regressions *p < 0.05

	Psychological intervention		Social intervention		Medication provided		Medication not available		Medication not affordable	
	Incidence rate ratio	p-value	Incidence rate ratio	p-value	Incidence rate ratio	p-value	Incidence rate ratio	p-value	Incidence rate ratio	p-value
Age groups										
0–14	1.03	0.26	1.20*	0.00090	1.04	0.22	1.22*	0.029	1.15	0.17
15–17	0.94*	0.019	0.84*	0.0028	0.94	0.083	1.06	0.66	0.94	0.55
18–24	1.00	0.89	1.02	0.70	0.98	0.35	1.01	0.87	1.00	0.96
25–34	0.99	0.61	0.96	0.18	0.99	0.48	0.86*	0.0095	1.01	0.86
35–44	0.99	0.66	0.91*	0.0062	0.99	0.55	0.72*	0.022	1.03	0.61
45–54	1.00	0.95	0.76*	0.0020	1.00	0.98	1.08	0.52	1.01	0.92
55–64	1.03	0.56	1.00	0.97	0.90*	0.040	0.92	0.69	0.94	0.67
65–74	0.96	0.55	1.02	0.90	1.04	0.63	0.99	0.97	0.94	0.69
75+	1.09	0.31	1.41	0.14	1.17	0.19	0.41	0.081	0.80	0.61
Unknown	1.07	0.36	1.24	0.19	1.09	0.36	0.60	0.17	0.86	0.61
Constant	0.68*	< 0.0001	0.27*	0	0.55*	< 0.0001	0.14*	< 0.0001	0.03*	0
N	231		230		231		190		183	
Sex										
Number of males	1.01	0.21	1.03	0.21	1.02	0.18	1.14*	0.046	1.10*	0.022
Number of females	0.98	0.057	0.96	0.069	0.97*	0.021	0.88*	0.034	0.97	0.31
Constant	0.66*	< 0.0001	0.17*	0	0.45*	0	0.10*	0	0.03*	0
N	206		205		206		165		157	
Freetown vs the rest										
Freetown (ref: the rest)	0.27*	0	0.24*	0.00050	0.16*	0	0.02*	< 0.0001	0.06*	< 0.0001
Constant	0.71*	0	0.26*	0	0.52*	0	0.13*	0	0.07*	0
N	236		235		236		192		184	
Ebola—districts with high endemicity vs the rest										
High Ebola endemicity districts (ref: the rest)	0.70*	0.0010	0.78	0.31	0.52*	< 0.0001	0.05*	< 0.0001	1.66	0.18
Constant	0.74*	< 0.0001	0.26*	0	0.57*	0	0.18*	0	0.05*	0
Observations	236		235		236		192		184	
2015 vs 2016										
2015 (ref: 2016)	1.24	0.090	1.40	0.25	0.91	0.59	3.09*	0.016	0.51	0.13
Constant	0.63*	< 0.0001	`	0	0.52*	< 0.0001	0.08*	0	0.09*	0
N	166		164		165		141		139	

The number of females was negatively associated with the number of psychotropics prescribed (p = 0.02). Other interventions were less strongly associated with sex.

The total number of prescriptions required was 3719. 24.7% (919 of 3719) were for medications that were inaccessible to the patient. The medication was unavailable in 52.8% (485) of these 919 cases, and unaffordable in 47.2% (434).

However, while no clinic was exempt from these problems, the few clinics with the highest numbers of contacts accounted for the majority of medication problems. For example, one MHU represented 74.0% (359) of all 485 cases of medication unavailability, which is 70.3% of all 511 prescriptions in that unit. Another MHU represented 43.3% (188) of the 434 cases in which patients could not afford medication, which is 40.6% of all 463 prescriptions in that clinic. Medication inaccessibility was less significant in clinics that had fewer contacts.

A total of 45 referrals were made to the SLPH. Two MHU—neither based in Freetown—referred 82.2% (37) of these. Three MHU recorded all 31 incidents of signposting to services other than the Hospital, which included referrals to international NGOs and other departments within the general hospital.

MHU staff conducted 2595 home visits, ranging from 1 to 49 per clinic per month. The median number of home visits per clinic per month was 6.0 (3.0–15.0).

### Ebola epidemic

A total of 327 Ebola survivors were seen over the 2-year period (80.1%, 262 in 2015 and 19.9%, 65 in 2016). One MHU saw 78.9% (258) of all Ebola survivors. There were a further 74 patients who had not contracted Ebola, but someone in their family had suffered from the virus.

Rates of ‘other’ (grief, trauma and stress, p = 0.001) and medically unexplained somatic complaints (p = 0.005) were significantly higher in the regions most affected by Ebola (Table 1).

### Variation by district

**Fig. 3:**
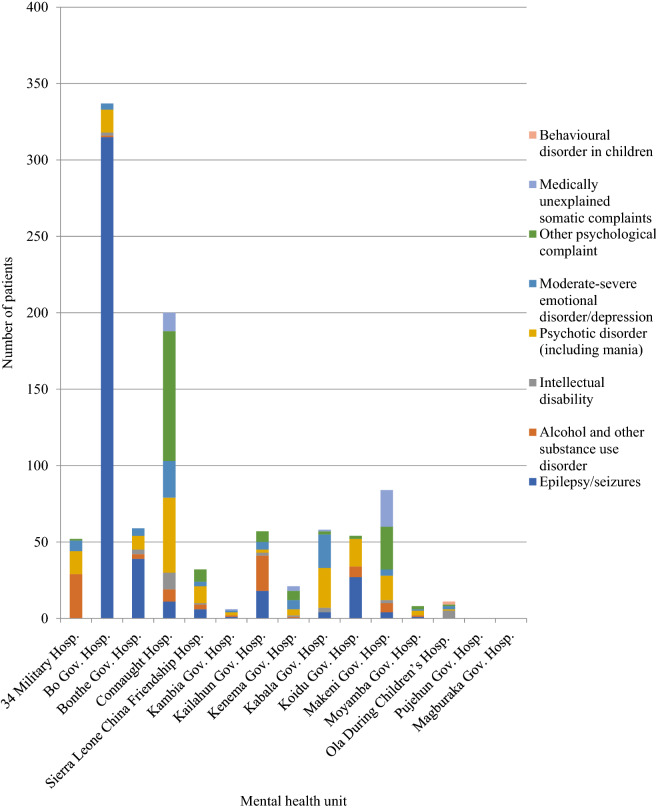
Referrals by diagnosis and mental health unit—number of referrals to all mental health units in 2015 and 2016; by diagnosis

There was variation in total numbers of new patients and each diagnosis across districts (Fig. 3). The diagnoses with the highest variability were epilepsy/seizures (ranging from 0.0 to 93.5%, which was 315 of 337 patients seen in one clinic over 2 years), alcohol and other substance abuse (0.0 to 55.8%, 29 of 52), and psychosis (0.0 to 18.4%, 216 of 1176).

Medication was significantly more available (p < 0.0001) and more affordable (p < 0.0001) in Freetown compared to other districts (Table 2).

### Variation by time

Of 2401 patients, 33.2% (798) presented in 2015, and 66.8% (1603) in 2016.

Depression (p < 0.0001) and medically unexplained symptoms (p = 0.0002) were significantly more associated with year 2015 than 2016.

The number of Ebola survivors decreased between 2015 (80.1%, 262 of 327 Ebola cases) and 2016 (19.9%, 65).

Medication unavailability was significantly associated with year 2015 compared to 2016 (p = 0.02, Table 2). There was no change in medication unaffordability over time.

### Treatment gap

Coverage by the MHUs (based on prevalence estimates from other studies) is 0.2% of the population experiencing schizophrenia and bipolar disorder, 0.2% with alcohol and substance use disorders, and 0.4% with epilepsy (Table 3) [1, 11, 22].

**Table 3:** Treatment gaps by diagnosis—estimated treatment gaps (proportion of untreated disease) by diagnosis category for epilepsy/seizures, substance use, psychosis, and depression, based on estimated prevalence data extrapolated from other studies

MHU, district	Region/district population	Diagnosis	No. patients treated with diagnosis	Reference data used to estimate expected prevalence			Est. no. people with diagnosis in district	Coverage (no. seen/no. with diagnosis, %)	Treatment gap (100-Coverage)
				Author, date	Study type	Prevalence			
All	7,396,000	Epilepsy/seizures	426	Ba-Diop A et al., 2014	Meta analysis	0.0143	105,762.8	0.40	99.6
		Alcohol and other substance use disorder	83	Charlston FJ et al., 2014	Estimates for West sub-Saharan Africa region	0.0052	38,459.2	0.22	99.8
		Intellectual disability	30	Alemu W et al., 2012	2002 Sierra Leone needs assessment post-civil war	0.01	73,960	0.04	100.0
		Psychotic disorder (including mania)	171	Charlston FJ et al., 2014	Estimates for West sub-Saharan Africa region	0.0114	84,314.4	0.20	99.8
		Moderate-severe emotional disorder/depression	84	Charlston FJ et al., 2014	Estimates for West sub-Saharan Africa region	0.0367	271,433.2	0.03	100.0
34 Military Hosp., Western Ar. Urban	1,055,964	Epilepsy/seizures	0	Ba-Diop A et al., 2014	Meta analysis	0.0143	15,100.3	0.00	100.0
		Alcohol and other substance use disorder	29	Charlston FJ et al., 2014	Estimates for West sub-Saharan Africa region	0.0052	5491.0	0.53	99.5
		Intellectual disability	0	Alemu W et al., 2012	2002 Sierra Leone needs assessment post-civil war	0.01	10,559.6	0.00	100.0
		Psychotic disorder (including mania)	15	Charlston FJ et al., 2014	Estimates for West sub-Saharan Africa region	0.0114	12,038.0	0.12	99.9
		Moderate-severe emotional disorder/depression	7	Charlston FJ et al., 2014	Estimates for West sub-Saharan Africa region	0.0367	38,753.9	0.02	100.0
		Total	51				81,942.8	0.06	99.9
Bo Gov. Hosp., Bo	575,478	Epilepsy/seizures	315	Ba-Diop A et al., 2014	Meta analysis	0.0143	8229.3	3.83	96.2
		Alcohol and other substance use disorder	1	Charlston FJ et al., 2014	Estimates for West sub-Saharan Africa region	0.0052	2992.5	0.03	100.0
		Intellectual disability	2	Alemu W et al., 2012	2002 Sierra Leone needs assessment post-civil war	0.01	5754.8	0.03	100.0
		Psychotic disorder (including mania)	15	Charlston FJ et al., 2014	Estimates for West sub-Saharan Africa region	0.0114	6560.4	0.23	99.8
		Moderate-severe emotional disorder/depression	4	Charlston FJ et al., 2014	Estimates for West sub-Saharan Africa region	0.0367	21,120.0	0.02	100.0
		Total	337				44,657.1	0.75	99.2
Bonthe Gov. Hosp., Bonthe	200,781	Epilepsy/seizures	39	Ba-Diop A et al., 2014	Meta analysis	0.0143	2871.2	1.36	98.6
		Alcohol and other substance use disorder	3	Charlston FJ et al., 2014	Estimates for West sub-Saharan Africa region	0.0052	1044.1	0.29	99.7
		Intellectual disability	3	Alemu W et al., 2012	2002 Sierra Leone needs assessment post-civil war	0.01	2007.8	0.15	99.9
		Psychotic disorder (including mania)	9	Charlston FJ et al., 2014	Estimates for West sub-Saharan Africa region	0.0114	2288.9	0.39	99.6
		Moderate-severe emotional disorder/depression	5	Charlston FJ et al., 2014	Estimates for West sub-Saharan Africa region	0.0367	7368.7	0.07	99.9
		Total	59				15,580.6	0.38	99.6
Connaught Hosp., Western Ar. Urban	1,055,964	Epilepsy/seizures	11	Ba-Diop A et al., 2014	Meta analysis	0.0143	15,100.3	0.07	99.9
		Alcohol and other substance use disorder	8	Charlston FJ et al., 2014	Estimates for West sub-Saharan Africa region	0.0052	5491.0	0.15	99.9
		Intellectual disability	11	Alemu W et al., 2012	2002 Sierra Leone needs assessment post-civil war	0.01	10,559.6	0.10	99.9
		Psychotic disorder (including mania)	49	Charlston FJ et al., 2014	Estimates for West sub-Saharan Africa region	0.0114	12,038.0	0.41	99.6
		Moderate-severe emotional disorder/depression	24	Charlston FJ et al., 2014	Estimates for West sub-Saharan Africa region	0.0367	38,753.9	0.06	99.9
		Total	103				81,942.8	0.13	99.9
Sierra Leone China Friendship Hosp., Western Ar. Rural	444,270	Epilepsy/seizures	6	Ba-Diop A et al., 2014	Meta analysis	0.0143	6353.1	0.09	99.9
		Alcohol and other substance use disorder	3	Charlston FJ et al., 2014	Estimates for West sub-Saharan Africa region	0.0052	2310.2	0.13	99.9
		Intellectual disability	1	Alemu W et al., 2012	2002 Sierra Leone needs assessment post-civil war	0.01	4442.7	0.02	100.0
		Psychotic disorder (including mania)	11	Charlston FJ et al., 2014	Estimates for West sub-Saharan Africa region	0.0114	5064.7	0.22	99.8
		Moderate-severe emotional disorder/depression	3	Charlston FJ et al., 2014	Estimates for West sub-Saharan Africa region	0.0367	16,304.7	0.02	100.0
		Total	24				34,475.4	0.07	99.9
Kambia Gov. Hosp., Kambia	345,474	Epilepsy/seizures	1	Ba-Diop A et al., 2014	Meta analysis	0.0143	4940.3	0.02	100.0
		Alcohol and other substance use disorder	1	Charlston FJ et al., 2014	Estimates for West sub-Saharan Africa region	0.0052	1796.5	0.06	99.9
		Intellectual disability	0	Alemu W et al., 2012	2002 Sierra Leone needs assessment post-civil war	0.01	3454.7	0.00	100.0
		Psychotic disorder (including mania)	2	Charlston FJ et al., 2014	Estimates for West sub-Saharan Africa region	0.0114	3938.4	0.05	99.9
		Moderate-severe emotional disorder/depression	1	Charlston FJ et al., 2014	Estimates for West sub-Saharan Africa region	0.0367	12,678.9	0.01	100.0
		Total	5				26,808.8	0.02	100.0
Kailahun Gov. Hosp., Kailahun	526,379	Epilepsy/seizures	18	Ba-Diop A et al., 2014	Meta analysis	0.0143	7527.2	0.24	99.8
		Alcohol and other substance use disorder	23	Charlston FJ et al., 2014	Estimates for West sub-Saharan Africa region	0.0052	2737.2	0.84	99.2
		Intellectual disability	2	Alemu W et al., 2012	2002 Sierra Leone needs assessment post-civil war	0.01	5263.8	0.04	100.0
		Psychotic disorder (including mania)	2	Charlston FJ et al., 2014	Estimates for West sub-Saharan Africa region	0.0114	6000.7	0.03	100.0
		Moderate-severe emotional disorder/depression	5	Charlston FJ et al., 2014	Estimates for West sub-Saharan Africa region	0.0367	19,318.1	0.03	100.0
		Total	50				40,847.0	0.12	99.9
Kenema Gov. Hosp., Kenema	609,891	Epilepsy/seizures	0	Ba-Diop A et al., 2014	Meta analysis	0.0143	8721.4	0.00	100.0
		Alcohol and other substance use disorder	1	Charlston FJ et al., 2014	Estimates for West sub-Saharan Africa region	0.0052	3171.4	0.03	100.0
		Intellectual disability	1	Alemu W et al., 2012	2002 Sierra Leone needs assessment post-civil war	0.01	6098.9	0.02	100.0
		Psychotic disorder (including mania)	4	Charlston FJ et al., 2014	Estimates for West sub-Saharan Africa region	0.0114	6952.8	0.06	99.9
		Moderate-severe emotional disorder/depression	6	Charlston FJ et al., 2014	Estimates for West sub-Saharan Africa region	0.0367	22,383.0	0.03	100.0
		Total	12				47,327.5	0.03	100.0
Kabala Gov. Hosp., Koinadugu	409,372	Epilepsy/seizures	4	Ba-Diop A et al., 2014	Meta analysis	0.0143	5854.0	0.07	99.9
		Alcohol and other substance use disorder	0	Charlston FJ et al., 2014	Estimates for West sub-Saharan Africa region	0.0052	2128.7	0.00	100.0
		Intellectual disability	3	Alemu W et al., 2012	2002 Sierra Leone needs assessment post-civil war	0.01	4093.7	0.07	99.9
		Psychotic disorder (including mania)	26	Charlston FJ et al., 2014	Estimates for West sub-Saharan Africa region	0.0114	4666.8	0.56	99.4
		Moderate-severe emotional disorder/depression	22	Charlston FJ et al., 2014	Estimates for West sub-Saharan Africa region	0.0367	15,024.0	0.15	99.9
		Total	55				31,767.3	0.17	99.8
Koidu Gov. Hosp., Kono	506,100	Epilepsy/seizures	27	Ba-Diop A et al., 2014	Meta analysis	0.0143	7237.2	0.37	99.6
		Alcohol and other substance use disorder	7	Charlston FJ et al., 2014	Estimates for West sub-Saharan Africa region	0.0052	2631.7	0.27	99.7
		Intellectual disability	0	Alemu W et al., 2012	2002 Sierra Leone needs assessment post-civil war	0.01	5061.0	0.00	100.0
		Psychotic disorder (including mania)	18	Charlston FJ et al., 2014	Estimates for West sub-Saharan Africa region	0.0114	5769.5	0.31	99.7
		Moderate-severe emotional disorder/depression	0	Charlston FJ et al., 2014	Estimates for West sub-Saharan Africa region	0.0367	18,573.9	0.00	100.0
		Total	52				39,273.4	0.13	99.9
Makeni Gov. Hosp., Bombali	606,544	Epilepsy/seizures	4	Ba-Diop A et al., 2014	Meta analysis	0.0143	8673.6	0.05	100.0
		Alcohol and other substance use disorder	6	Charlston FJ et al., 2014	Estimates for West sub-Saharan Africa region	0.0052	3154.0	0.19	99.8
		Intellectual disability	2	Alemu W et al., 2012	2002 Sierra Leone needs assessment post-civil war	0.01	6065.4	0.03	100.0
		Psychotic disorder (including mania)	16	Charlston FJ et al., 2014	Estimates for West sub-Saharan Africa region	0.0114	6914.6	0.23	99.8
		Moderate-severe emotional disorder/depression	4	Charlston FJ et al., 2014	Estimates for West sub-Saharan Africa region	0.0367	22,260.2	0.02	100.0
		Total	32				47,067.8	0.07	99.9
Moyamba Gov. Hosp., Moyamba	318,588	Epilepsy/seizures	1	Ba-Diop A et al., 2014	Meta analysis	0.0143	4555.8	0.02	100.0
		Alcohol and other substance use disorder	1	Charlston FJ et al., 2014	Estimates for West sub-Saharan Africa region	0.0052	1656.7	0.06	99.9
		Intellectual disability	0	Alemu W et al., 2012	2002 Sierra Leone needs assessment post-civil war	0.01	3185.9	0.00	100.0
		Psychotic disorder (including mania)	3	Charlston FJ et al., 2014	Estimates for West sub-Saharan Africa region	0.0114	3631.9	0.08	99.9
		Moderate-severe emotional disorder/depression	1	Charlston FJ et al., 2014	Estimates for West sub-Saharan Africa region	0.0367	11,692.2	0.01	100.0
		Total	6				24,722.4	0.02	100.0
Ola During Children’s Hosp., Western Ar. Urban	1,055,964	Epilepsy/seizures	0	Ba-Diop A et al., 2014	Meta analysis	0.0143	15,100.3	0.00	100.0
		Alcohol and other substance use disorder	0	Charlston FJ et al., 2014	Estimates for West sub-Saharan Africa region	0.0052	5491.0	0.00	100.0
		Intellectual disability	5	Alemu W et al., 2012	2002 Sierra Leone needs assessment post-civil war	0.01	10,559.6	0.05	100.0
		Psychotic disorder (including mania)	1	Charlston FJ et al., 2014	Estimates for West sub-Saharan Africa region	0.0114	12,038.0	0.01	100.0
		Moderate-severe emotional disorder/depression	2	Charlston FJ et al., 2014	Estimates for West sub-Saharan Africa region	0.0367	38,753.9	0.01	100.0
		Total	8				81,942.8	0.01	100.0
Pujehun Gov. Hosp., Pujehun	346,461	Epilepsy/seizures	0	Ba-Diop A et al., 2014	Meta analysis	0.0143	4954.4	0.00	100.0
		Alcohol and other substance use disorder	0	Charlston FJ et al., 2014	Estimates for West sub-Saharan Africa region	0.0052	1801.6	0.00	100.0
		Intellectual disability	0	Alemu W et al., 2012	2002 Sierra Leone needs assessment post-civil war	0.01	3464.6	0.00	100.0
		Psychotic disorder (including mania)	0	Charlston FJ et al., 2014	Estimates for West sub-Saharan Africa region	0.0114	3949.7	0.00	100.0
		Moderate-severe emotional disorder/depression	0	Charlston FJ et al., 2014	Estimates for West sub-Saharan Africa region	0.0367	12,715.1	0.00	100.0
		Total	0				26,885.4	0.00	100.0
Magburaka Gov. Hosp., Tonkolili	531,435	Epilepsy/seizures	0	Ba-Diop A et al., 2014	Meta analysis	0.0143	7599.5	0.00	100.0
		Alcohol and other substance use disorder	0	Charlston FJ et al., 2014	Estimates for West sub-Saharan Africa region	0.0052	2763.5	0.00	100.0
		Intellectual disability	0	Alemu W et al., 2012	2002 Sierra Leone needs assessment post-civil war	0.01	5314.4	0.00	100.0
		Psychotic disorder (including mania)	0	Charlston FJ et al., 2014	Estimates for West sub-Saharan Africa region	0.0114	6058.4	0.00	100.0
		Moderate-severe emotional disorder/depression	0	Charlston FJ et al., 2014	Estimates for West sub-Saharan Africa region	0.0367	19,503.7	0.00	100.0
		Total	0				41,239.4	0.00	100.0

## Discussion

This study is the first insight since 2002 into people with mental health problems accessing services in Sierra Leone and show that the treatment gap remains high.

### Patient characteristics

Men and women accessed services equally. The number of males was associated with numbers with psychosis, depression and substance abuse. The largest age category was 25–34 years (23.4%). However, globally half of mental health disorders start by the mid-teens and three quarters by the mid-twenties, and in Sierra Leone 48.3% of the population is aged under 18 [23, 24]. This emphasises the need for robust systems that are suitable for younger people.

Epilepsy/seizures accounted for the highest proportion of diagnoses seen. This is unlikely to indicate a greater prevalence amongst the general population compared to mental health conditions, but may reflect improved referral mechanisms and health literacy resulting from organisations including the Epilepsy Association of Sierra Leone. An estimated 1.43% of the population in West Africa have active epilepsy [22]. There is only one neurologist in Sierra Leone, and MHUs may be playing an important role in reducing the treatment gap [11].

Psychosis was the most frequently diagnosed mental health disorder, surpassing more common disorders such as depression (17.5% and 8.6% of MHU patients respectively).

The under-representation of depression in newly established services replicates studies elsewhere [25]. The comparative population prevalence rates for psychosis and depression are 1.14% and 3.67% respectively in Western Africa [1]. It is possible that patients with less visible disorders such as depression may not prompt the same level of concern as psychotic disorders, that there may be differences in perceptions about treatment, and that affective disorders are not viewed in the same explanatory model as psychotic disorders [26, 27]. Whilst we expect the rates of depression to be much higher, it is important to consider factors that promote resilience such as social support through community acceptance [28, 29].

The 327 Ebola survivors attending MHU represent 3.3–6.9% of the total survivors (4750 laboratory-confirmed, 10,000 estimated) [30, 31]. Studies in Ebola sequelae show prevalence rates of 10.9–49% for depression, and up to 34% for post-traumatic stress disorder [32–36]. The MHU appear to be helping reduce the treatment gap for mental health disorders among Ebola survivors, particularly for people with grief, stress and trauma, and medically unexplained somatic complaints. The majority presented in 2015 (80.1%) compared to 2016 (19.9%), indicating mental health problems may present as early sequelae of Ebola [19]. However, this result may be affected by targeted health initiatives to screen Ebola survivors mental health problems. This could explain the differences in data between districts, which are not fully accounted for by endemicity.

Low numbers of reported suicide and self-harm attempts in this study (33 in 2 years) could be further explored to evaluate if this is an issue of reporting or reflects a true picture. The WHO estimates the suicide rate in Sierra Leone is 9.7 per 100 000, higher than the sub-Saharan average of 7.5 [37]. This would equate to 733.0 per year in Sierra Leone, and for every completed suicide there are 50 attempts [38]. Among psychiatry patients the suicide attempt rate is 15–50% [39]. The results from this study should be interpreted in a context of wider social, cultural and stigma related issues around suicide in Sierra Leone.

### Patient numbers and pathways to care

The increase in service uptake by year is likely a reflection of service establishment due to the successive opening of MHU. The last MHU (the CAMH Unit) opened in March 2016.

There is wide variation in the number of referrals into different MHU. These differences appear un-related to district population and should be investigated further. Issues around staffing levels, geography, community access to mental health information, levels of education, traditional beliefs, stigma, and local opportunities to align resources towards mental health and epilepsy are likely contributing factors. These factors also might help explain the geographic variation in the types of disorders presenting to services.

The most common referral sources are self-referrals and referrals from other general hospital departments. A wide range of organisations are aware of, and referring to, the MHU. There are no data on how self-referred patients heard about the service. Possibilities include radio campaigns, word-of-mouth and healthcare professionals. Most patients are outpatients, indicating these services are making treatment available to people living in their homes.

### Mental health treatments

Among clinics with the highest numbers of contacts there are sizeable problems with accessing medicines. These problems transcend all patient ages and sexes. Medication inaccessibility potentially undermines quality of clinical care and trust in the service. This highlights the need for the MOHS to include psychotropic medications in procurement plans, to strengthen supply chain mechanisms and prevent leaks within the supply [40]. It is possible that some patients accessing MHU may have been taking medication prior to 2015. However, access to psychotropic medication in the country is scarce, except for unreliable supplies within the Psychiatric Hospital [41].

Whilst supply is more accessible in the capital city, medication often remained unaffordable. Innovative means of mental health financing may be required.

Few other psycho-social services are available, aligning with the low rates of signposting to other services. Treatment and rehabilitation frequently require the involvement of family and community, therefore the most common interventions applied are likely to be psychoeducation and attempts to engage wider supports. These activities are captured in the number of social and psychological interventions. Psychological interventions beyond psychoeducation would mostly have consisted of counselling and problem-solving therapy, which is appropriate to the level of training of MHU staff. Referrals from the MHU to the Psychiatric Hospital are low (45 in 2 years). It is possible that the nurses felt adequately prepared to handle most cases; patients or their families blocked the referral due to the challenges associated with travel, or were against admission for other factors including the hospital being under-resourced; or there was a preference for alternative forms of care. Since deinstitutionalisation, psychiatric inpatient facilities only provide 7% of psychiatric care globally [42].

Home visits formed a significant part of the workload. Home visits may be preferred by patients because of travel difficulties or stigma associated with the MHU or may be the only option to see people who are very unwell. As much as this is a good indicator of patient-centred care uptake, the increasing demand on mental health nurses’ time poses a risk towards the quality of services.

### Treatment gap

Despite the positive trend in service uptake, coverage by the MHUs for the two most common disorders, epilepsy and psychosis, are only 0.4% and 0.2% respectively. This may slightly reduce the treatment gap for mental health disorders, which in 2009 was 98.0% (the remaining 2.0% treated by the Psychiatric Hospital and NGOs) [11]. In 2016, the treatment gap for childhood disorders was estimated at over 99.8% [43]. This demonstrates that services are still not meeting the needs of the population and further exploration of how to develop services is warranted. This is especially important given that the population has risen—by 2 million since the most recent epidemiological survey for mental health in 2002—and the burden of mental health disorders is expected to greatly increase over the next decade [1].

### Challenges in service delivery

Efforts to increase service uptake are threatened by a multitude of challenges, ranging from problems with the wider health system to issues specific to mental health. These include: human resource constraints, inadequate medication supply chains and the availability of only one psychiatric inpatient facility. Of the 15 MHUs, 13 are each staffed by one nurse working alone. These staff are responsible for all clinical and service management. Staff ill-health can result in no patients being seen.

Mental health nurses are not fully integrated into the health system [21]. They receive low remuneration for their work and have limited opportunities for career progression, potentially impacting future attrition rates. There is limited potential for the MOHS to conduct training or supportive supervision, both of which are provided by NGOs. The MOHS has no specific budget for mental health outside SLPH, which has its own funding difficulties.

Patient-related factors that may have contributed to low rates of presentation at MHU include stigma, low mental health literacy, preference for traditional/faith-based healers, and inability to finance health cost highlighted by the number of patients who could not afford medications [7].

### Wider efforts in task sharing

Through the support of partners the roll out of mental health services to primary care has led to training in Psychological First Aid and mhGAP intervention guidelines to general nurses, Community Health Officers and medical officers; aiming to improve mental health literacy, task-sharing and collaborative decision making [20, 44]. This initiative still needs active coordination and division of roles and responsibilities.

The MOHS has launched the new Mental Health Policy and Strategic Plan 2019–2023. Targets for training more mental health professionals are included. Research and M&E are at the forefront of the policy. As yet finance options are uncertain.

### Limitations

The results are limited by the accuracy and completeness of the data. It is possible that missing data introduced bias into the results. For example, missing data on diagnoses could reflect recording errors, but may have resulted from staff struggling to reach diagnoses, which could be biased towards particular diagnoses. Certain clinics (with high numbers of new patients) contributed a greater proportion of missing data, possibly indicating capacity issues within these MHU around data collection. Issues with routine collection of health data in Sierra Leone are certainly not limited to mental health [31, 32]. M&E systems in sub-Saharan Africa are challenged by limited human and financial resources, weak information systems, and limited demand for M&E [45]. MHU clinical staff were responsible for the reporting of data used in this study, and many had only received limited training and support on M&E. As a result of this study, further training has been conducted to improve staff skills and capacity around data collection.

The summary reports in this study use diagnoses and treatments according to the broad categories of the mhGAP. Psychosis, for example, includes schizophrenia and bipolar disorder, which have different prognoses. It was not possible to assess the diagnoses for reliability. However, mhGAP was developed by experts in the field as a viable method of scaling up mental health treatment using non-specialist services [46]. The definition of some variables like psychological intervention and social intervention was not distinct. Data on individual medications were not available.

Despite data quality issues, this study is valuable due to the novel nature of the intervention and current paucity of data on mental health service provision and burden of mental health disorders in Sierra Leone. With limited funding for such research, the use of routine data provides an opportunity to gain insights on service provision and disease burden to inform decision-making. Integration of mental health data into the routine government M&E systems could promote improve data quality and availability in the longer term.

## Conclusions

In resource-poor environments the roll-out of decentralised nurse-led MHU such as these new services in Sierra Leone can provide vital treatment, particularly to younger people, and people with epilepsy, psychosis, and psychological symptoms associated with stress, grief and trauma. The rate of depression was lower than expected indicating services were not meeting need in this area. The numbers of referrals and subsequent interventions increased during the study period but there is a need to further improve coverage and medication accessibility. The variation in the provision of clinical services warrants further exploration. This study addresses a research gap for the service utilisation of specialised nurse-led MHU in sub-Saharan Africa.

The results of this study have been disseminated to policy makers and clinical staff in Sierra Leone, to support the development of recommendations on training, mentorship, data collection and use. Findings were used to inform and advocate for the approval of the recently launched MOHS Mental Health Policy and Strategic Plan 2019–2023, which includes targets for training more mental health professionals and improving research capacity and M&E. Further evaluation following the rollout of this plan will help to identify progress and bottlenecks to the provision of high-quality mental health services.

## Data Availability

The datasets analysed during the current study are available from the corresponding author on reasonable request.

## Appendices

### Appendix 1 District Mental Health Unit Monthly Data Form

.

### Appendix 2

See Table

**Table 4:** Missing data—the number and proportion (%) of data entries that were complete and accurate, by category of the data sheet and by MHU (*report availability indicates that the MHU was operational; reports not available for months when clinics were not open)

Mental Health Unit	Total no. of monthly reports*	Reports available (complete and accurate) according to categories N (%)											
		Sex	Age	Diagnosis	Ebola survivors	New patients	Referral sources	Psychotropic medication	Psychological intervention	Social intervention	Medication not available	Medication not affordable	Total availability by MHU
34 Military Hospital	22	22 (100.0)	22 (100.0)	22 (100.0)	22 (100.0)	22 (100.0)	22 (100.0)	22 (100.0)	22 (100.0)	22 (100.0)	22 (100.0)	22 (100.0)	242 (100.0)
Bo Government Hospital	23	1 (4.3)	20 (87.0)	23 (100.0)	23 (100.0)	23 (100.0)	22 (95.7)	23 (100.0)	23 (100.0)	23 (100.0)	23 (100.0)	23 (100.0)	227 (89.7)
Bonthe Government Hospital	23	18 (78.3)	9 (39.1)	23 (100.0)	23 (100.0)	21 (91.3)	23 (100.0)	13 (56.5)	23 (100.0)	20 (87.0)	14 (60.9)	15 (65.2)	202 (79.8)
China-Sierra Leone Friendship Hospital (Jui)	20	15 (75.0)	14 (70.0)	12 (60.0)	19 (95.0)	19 (95.0)	14 (70.0)	19 (95.0)	19 (95.0)	19 (95.0)	14 (70.0)	8 (40.0)	172 (78.2)
Connaught Hospital	22	22 (100.0)	22 (100.0)	14 (63.6)	22 (100.0)	22 (100.0)	22 (100.0)	22 (100.0)	22 (100.0)	20 (90.0)	0 (0.0)	0 (0.0)	188 (77.7)
Kabala Government Hospital	22	22 (100.0)	22 (100.0)	22 (100.0)	22 (100.0)	22 (100.0)	21 (95.5)	22 (100.0)	22 (100.0)	0 (0.0)	22 (100.0)	22 (100.0)	219 (90.5)
Kailahun Government Hospital	16	16 (100.0)	16 (100.0)	15 (93.8)	16 (100.0)	16 (100.0)	12 (75.0)	16 (100.0)	16 (100.0)	12 (75.0)	15 (93.8)	15 (93.8)	165 (93.8)
Kambia Government Hospital	8	8 (100.0)	8 (100.0)	8 (100.0)	8 (100.0)	8 (100.0)	7 (87.5)	7 (87.5)	8 (100.0)	0 (0.0)	6 (75.0)	4 (50.0)	72 (81.8)
Kenema Government Hospital	24	1 (4.2)	1 (4.2)	1 (4.2)	21 (87.5)	21 (87.5)	7 (29.2)	14 (58.3)	13 (54.2)	7 (29.2)	13 (54.2)	12 (50.0)	111 (42.0)
Koidu Government Hospital	19	11 (57.9)	6 (31.6)	8 (42.1)	19 (100.0)	17 (89.5)	6 (31.6)	19 (100.0)	17 (89.5)	6 (31.6)	5 (26.3)	5 (26.3)	119 (56.9)
Magburaka Government Hospital	24	6 (25.0)	5 (20.8)	4 (16.7)	24 (100.0)	24 (100.0)	23 (95.8)	19 (79.2)	22 (91.7)	7 (29.2)	21 (87.5)	21 (87.5)	176 (66.7)
Makeni Government Hospital	20	20 (100.0)	20 (100.0)	20 (100.0)	20 (100.0)	20 (100.0)	19 (95.0)	5 (25.0)	15 (75.0)	1 (5.0)	18 (90.0)	18 (90.0)	176 (80.0)
Moyamba Government Hospital	12	5 (41.7)	4 (33.3)	4 (33.3)	11 (91.7)	12 (100.0)	7 (58.3)	8 (66.7)	11 (91.7)	2 (16.7)	6 (50.0)	6 (50.0)	76 (57.6)
Ola During Children’s Hospital	10	0 (0.0)	9 (90.0)	2 (20.0)	10 (100.0)	10 (100.0)	9 (90.0)	0 (0.0)	10 (100.0)	9 (90.0)	10 (100.0)	10 (100.0)	79 (71.8)
Pujehun Government Hospital	20	10 (50.0)	10 (50.0)	1 (5.0)	19 (95.0)	20 (100.0)	16 (80.0)	18 (90.0)	19 (95.0)	20 (100.0)	13 (65.0)	13 (65.0)	159 (72.3)
Total availability by category	285	177 (62.1)	188 (66.0)	179 (62.8)	279 (97.9)	277 (97.2)	230 (80.7)	227 (79.6)	262 (91.9)	168 (58.9)	202 (70.9)	194 (68.1)	

4.
